# Preparation and *In-vitro* Evaluation of Rifampin-loaded Mesoporous Silica Nanoaggregates by an Experimental Design

**Published:** 2015

**Authors:** Meysam Mohseni, Kambiz Gilani, Zohreh Bahrami, Noushin Bolourchian, Seyed Alireza Mortazavi

**Affiliations:** a *Department of Pharmaceutics, School of Pharmacy, Shahid Beheshti University of Medical Sciences, Tehran, Iran.*; b *Department of Pharmaceutics, School of Pharmacy, Tehran University of Medical Sciences, Tehran, Iran.*; c *School of Chemistry, College of Science, University of Tehran, Tehran, Iran. *

**Keywords:** Spray drying, Rifampin, Experimental design, Silica nanoaggregates, Drug release

## Abstract

The goal of this research is preparation, optimization and *in-vitro* evaluation of rifampin-loaded silica nanoparticles in order to use in the pulmonary drug delivery. Nanoparticles are exhaled because of their small size. Preparation of nanoaggregates in a micron-size scale and re-dispersion of them after deposition in the lung is an approach to overcome this problem. We used this approach in our research. Rifampin was selected as a model lipophilic molecule since it was a well-documented and much used anti tuberculosis drug. A half factorial design was used to identify significant parameters of the spray drying process. The results showed that feed concentration, feed pH and the interaction between feed flow rate and gas atomizer flow rate had statistically significant effects on the particle size of nanoaggregates. The Box-Behnken design was employed to optimize the spray drying process. Finally, a quadratic equation which explains the relation between independent variables and aerodynamic diameter of nanoaggregates was obtained. Rifampin-loaded silica nanoaggregates underwent different *in-vitro* tests including: SEM, Aerosol performance and drug release. The *in-vitro* drug release was investigated with buffer phosphate (pH=7.4). Regarding the drug release study, a triphasic pattern of release was observed. The rifampin-loaded silica nanoaggregates were capable of releasing 90% drug content after 24 h in combination patterns of release. The prepared rifampin-loaded nanoaggregates seem to have a potential to be used in a pulmonary drug delivery.

## Introduction

Since drug-loaded particles are suitable for the controlled release and drug targeting, they have been the focus of researches on drug delivery systems (1). Developments in the encapsulation technology have allowed the preparation of a large range of submicron-sized drug loaded particles. These nanoparticles may have widespread potential as drug carriers due to the presence of an organic shell or to their organization (colloidal systems, liposomes, microemulsion, *etc*.) (1-5). Among these drug delivery systems, inorganic porous materials are emerging as a new category of host/guest systems. Due to some interesting features such as their biological stability and drug-releasing properties (6), there is a significant and increasing interest in these potential carriers. Several porous minerals have been used including synthetic zeolithe, silica xerogel material and porous ceramic (6-8). In comparison to organic-based drug delivery vehicles, drug-bearing silica particles offer several advantages. (1) They are more stable to temperature and pH fluctuations, (2) their morphology can be precisely controlled to manipulate the drug targeting-ability and its release profile, and (3) their surface, just like other metal oxides, is decorated with hydroxyl groups making the silica particles less vulnerable to the opsonization by the body immune system (9). In particular, Mesoporous silica nanoparticles (MSN) offer several attractive features, such as a large surface area, easily modified pore size and volume, as well as being chemically inert and allowing easier functionalization of their surface (10-19). All these features allow better control of drug loading and release. Administration of MSNs can take place through parenteral and oral route. One of the main advantages is the ability to increase the solubility of poorly water soluble drugs while they can also be used for hydrophilic active agents. Thus, high drug loading can be achieved with loading capacities normally varying from 10 to 34% (20) or up to 60% in extreme cases (21). They have been also used for controlled release and drug targeting providing sustained release for 16 h (22).

There has always been a concern for silica nanoparticles toxicity, but many studies have shown that this concern is undervalued. For example one study on early life stage of Zebrafish has shown that silica nanoparticles and/or aggregates mainly accumulate on the chorion of embryos and exhibit no overt emryotoxicity (23), another study has shown that silica nanoparticles do not reduce glutathione level nor generating ROS in mouse keratinocytes (24). Among the various drug delivery systems considered for pulmonary application, nanoparticles demonstrate several advantages for the treatment of respiratory diseases, like prolonged drug release, cell specific targeted drug delivery or modified biological distribution of drugs, both at the cellular and organ level (25), another study has shown single and repeated dose in intravenously exposed mouse cause no death (26).

Rifampin is an antibiotic against Mycobacterium Tuberculosis which is widely used for treatment of Tuberculosis, because of its poor bioavailability pulmonary drug delivery could be considered as an alternative route for rifampin administration. In our previous work, we prepared rifampin-loaded nanosilica particles as a potential system for pulmonary drug delivery (27). Nevertheless, direct inhalation of the nanoparticles is not

plausible because nanoparticulate aerosols are predominantly exhaled from the lung due to their extremely small inertia. In inhaled drug delivery, the particle aerodynamic diameter (*d*A) is used to characterize the distance travelled by the inhaled particles in the human respiratory airways. Spherical particles with large *d*A (>10 µm) tend to deposit in the mouth and throat regions, whereas particles with small *d*A (<1 µm) remain suspended in the air flow and exhaled from the lung (28). Particles for inhaled drug delivery are therefore designed with *d*A ≈2–4 µm to facilitate their deposition in the lung alveolar and bronchial regions. As nanoparticles possess dA<1 µm due to the small dG, they must be formulated into a micron-scale structure with dA ≈2–4 µm to facilitate their delivery to the lung by inhalation. One formulation approach is by spray drying the nanoparticulate suspension with pharmaceutical excipients (*e.g*. lactose, chitosan) to produce micron-size particles with dA ≈ 2–4 µm in which the drug-bearing nanoparticles are encapsulated within the excipient particles (29, 30). The drawback of this formulation approach is that the therapeutic efficacy of the nanoparticles depends on the dissolution rate of the excipient particles. Furthermore, excipient particles in dG about 2–4µm range are typically very cohesive resulting in poor aerosolization efficiency. As a result, the spray-dried particles must be blended with coarser particles (*d*G >50 µm) to facilitate their aerosolization. An alternative formulation approach is to spray dry the nanoparticulate suspension into large spherical nano-aggregates, which disassociate back into the primary nanoparticles in the lung interstitial fluid to perform their therapeutic functions (31, 32). The large *d*G of the nano-aggregates (>5 µm) reduces their tendency to agglomerate resulting in high aerosolization efficiency, whereas the low ρ_eff_ attributed to the porous structure results in geometrically large particles having *d*A ≈2–4 µm that is ideal for inhalation delivery. 

The objectives of the present work are (1) to identify the spray-drying formulation parameters that govern the Rifampin-loaded silica nano-aggregate morphology, and (2) to optimize the spray-drying formulation parameters with an aim to improve the particle size distribution of the nano-aggregates and (3) *in-vitro* evaluation of Rifampin-loaded nanoaggregates.

## Experimental

*Materials*

Rifampin-loaded nanoparticles were prepared and characterized based on our previous study (27) which have a Rifampin loading of 61 % and the Z-average of 290 nm. Hydrogen chloride, potassium hydroxide and sodium hydrogen phosphate (dibasic) were obtained from Merck (Germany). All chemicals were used as received.

Büchi B-290 Mini Spray Dryer is operated in the present work in an open-loop mode using air as the drying gas as no flammable solvent is used in the nanoparticulate suspension preparation, the open-loop operation mode is preferred because it is more stable and more cost-effective compared to the close-loop mode. Importantly, the operation mode of the spray dryer, which influences the drying rate, has been shown to significantly affect the resulting particle morphology (33). The adjustable spray-drying parameters are (1) the inlet drying temperature, (2) the gas atomizing flow rate, (3) the feed rate, and (4) the feed concentration. The nozzle diameter of the two-fluid flow atomizer is 1.5 mm.

In the screening design, a half-factorial design involving five experimental variables (*i.e*. 2^(5−1)^ design) is conducted to identify the spray-drying formulation parameters that have significant influences on the nano-aggregate dA. Two independent replicates are obtained for each experimental run. The 2^(5−1)^ half-factorial design is a Resolution V design in which no main effects (*e.g*. A, B, C) or second order interaction effects (*e.g*. AB, AC, BC) are confounded with each other. The statistical analysis is conducted using Design-Expert version 7 statistical software (Stat-Ease Inc., USA), where third order interactions and higher are neglected. After the screening design, a response surface methodology by Box-Behnken design involving only the significant parameters are conducted to optimize the formulation parameters. The five parameters investigated in the screening design are (A) the feed concentration, (B) the nanoparticulate suspension pH, (C) the inlet temperature, (D) the feed rate, and (E) the gas atomizing flow rate. The similarity in the magnitude of the surface tension and the viscosity of the colloidal silica suspension with respect to water, and the fact that the magnitude does not significantly vary over a wide range of conditions (*i.e*. pH, concentration), suggest that the influences of these two particular properties on the resulting nanoaggregate morphology obtained from different formulation parameters are likely to be insignificant. The effect of the suspension pH nevertheless still needs to be examined as it influences the colloidal silica stability that governs the shell buckling. In this regard, the colloidal silica suspension is most stable near their isoelectric point (pH≈2–3), and becomes less stable at a higher pH up to pH≈6, before it re-enters a high-stability region between pH≈8 and 10.5 (34). Several studies on particle production by spray drying have primarily identified the drying temperature and the feed rate as the two spray drying parameters that exhibit the most influence on the resulting particle morphology (-). More specifically, the drying gas velocity was found to be the most significant parameter governing the production of silica micro-aggregates in a fluidized bed granulator (38, 39). Whether similar results are obtained in the production of silica nano aggregates are to be examined in the present work.

*In-vitro evaluation of rifampin-loaded mesoporous silica nanoaggregates*

*SEM*

The morphology of the prepared samples was characterized using a scanning electron microscopy (SEM). The samples were attached to aluminum stubs with double side adhesive carbon tape then gold coated and examined using a scanning electron microscope (SEM, LEO 1455VP, Cambridge, U.K.).

***Particle size measurements***

The particle size of the nanoaggregates was measured with Malvern Mastersizer 2000 (Malvern, UK). The analysis was performed at a temperature of 25 °C and by dry method. For calculation of dA the below equation was used.

 Equation (1) dA=dG ρ effρ unity2

Where dG is the particle geometric size (obtained from Mastersizer 2000), ρ_unity_ is equal to 1 g/cm^3^, and ρ_eff_ is the particle effective density defined as the particle mass divided by its total volume including the open and closed pores. The effective particle densities are determined by pcynometer and tap densitometer (Quantachromme, USA), respectively. The tap density is measured after 2000 taps using three different samples of 4 mL each. The measured tap density is corrected by a factor of 0.79^−1^ to obtain ρ_eff_ by taking into account the imperfect packing after tapping (40). 

*HPLC*

To assay Rifampin, HPLC system was used. The obtained samples from release test were injected to HPLC system. Rifampin was determined using HPLC analysis with UV detection at 254 nm. Rifampin was analyzed by a Knauer HPLC system consisting of a 1000 pump and a 2500 UV–VIS detector (Germany). Analysis was carried out on a Nucleodur C8 column (150×4.6 mm, 5 μm). The mobile phase consisted of a 66:34 (%v/v) mixture of phosphate buffer and acetonitrile, the flow rate was 1.5 mL/min and the detection wavelength was 254 nm. The injection volume was 10 μL. 

*In-vitro drug release*

The *in-vitro* drug release study was carried out using a dialysis bag (cellulose membrane ,MW cut-off 12,400, Sigma–Aldrich), which (1) allows free diffusion of the drug molecules into the release medium, while at the same time (2) completely separates the nanoparticles from the release medium. About 10 mg of the Rifampin-loaded nanoaggregates were suspended in 2 mL of phosphate buffer solution (pH 6.8) inside a dialysis bag. The pH of the buffer solution was adjusted to 7.4. The dialysis bag was then placed in 38 mL of the buffer solution (sink condition) at 37 °C under magnetic stirring. At successive time intervals, aliquots (2 mL) of the release medium was collected and replaced with a fresh buffer solution. The collected sample was next analyzed using the HPLC. The *in-vitro* drug release was carried out for 24 h. Each experiment was conducted in triplicate. This test was also performed on the Rifampin and physical mixture of Rifampin and silica nanoparticles (41).

*Aqueous re-dispersibility characterizations*

To quantify the mass percentage of the re-dispersed nanoaggregates, 10 mg of the nanoaggregates are dispersed in 2 mL of deionized water and the suspension is let sit for 30 min under occasional stirrings. Afterwards, the size of the particles present in the suspension is analyzed by Zetasizer (Malvern, UK) to determine the extent of the re-dispersion, Each experiment was conducted in triplicate at 37 °C. (42).

*Aerosol performance of Rifampin-loaded nanoaggregates*

The *in-vitro* pulmonary deposition of the powders was determined by Anderson cascade impactor (ACI) from Copley Scientific (UK). A dry powder formulation device, Novartis Cyclohaler® (Switzerland), was filled with a hard gelatin capsule loaded with 10 mg of each formulation. Once the assembly had been checked to be tight, the Cyclohaler® had been inserted to the rubber mouthpiece attached to the throat part of the ACI. The test was operated at 50 L/min for 5 s. The flow rate was achieved using a rotary vein pump from Copley Scientific (UK). After the operation, the ACI components had been washed into separate volumetric with water. Their contents were assayed for Rifampin by HPLC. Fine particle dose (FPD) was considered as the amount of drug deposited in stage 2 (dA < 4.4 μm). The emitted dose (ED) was determined as a percent of total powder exiting the capsule and the device. The FPF was calculated as the percent of the ratio of FPD to the total amount of drug emitted per capsule.

## Results and Discussion

*Nanoaggregates preparation*

*Identifying significant spray drying parameters*

The 22 experimental runs of the half-factorial design and their responses are presented in Table 1. The results in Table 1 are the average of the two independent replicates. Preliminary runs are first conducted to establish the feasible range for each formulation parameter. The preliminary runs indicate that the feed rate and the gas atomizing flow rate must be selected to prevent excessive wetting of the glass chamber by the spray droplets.

**Table 1:** Summary of the 2^(5-1)^ half factorial experimental design for screening the significant factors of spray drying process

**Run**	**Block**	**Factor 1**	**Factor 2**	**Factor 3**	**Factor 4**	**Factor 5**	**Response**
		**A:Concentration** **(w/w%)**	**B:pH**	**C:T** **°C**	**D:Feed** **mL/min**	**E:Air flow** **m** ^3^ **/h**	**dA** **µm**
1	Week 1	4	9	110	1	0.378	6.2
2	Week 1	2	9	110	2	0.343	2.2
3	Week 1	4	9	90	1	0.343	5.8
4	Week 1	2	9	90	2	0.378	2.1
5	Week 1	3	7	100	1.5	0.361	2.4
6	Week 1	3	7	100	1.5	0.361	2.8
7	Week 1	4	5	110	2	0.343	2.3
8	Week 1	2	5	90	1	0.343	3.1
9	Week 1	2	5	110	1	0.378	2.6
10	Week 1	4	5	90	2	0.378	2.8
11	Week 1	3	7	100	1.5	0.361	2.9
12	Week 2	4	9	90	2	0.343	4.3
13	Week 2	3	7	100	1.5	0.361	2.7
14	Week 2	3	7	100	1.5	0.361	2.6
15	Week 2	4	9	110	2	0.378	6.5
16	Week 2	4	5	110	1	0.343	3.5
17	Week 2	4	5	90	1	0.378	1.5
18	Week 2	2	9	110	1	0.343	1.9
19	Week 2	2	5	90	2	0.343	4.1
20	Week 2	2	9	90	1	0.378	1.2
21	Week 2	3	7	100	1.5	0.361	2.9
22	Week 2	2	5	110	2	0.378	3.9

**Figure1:**
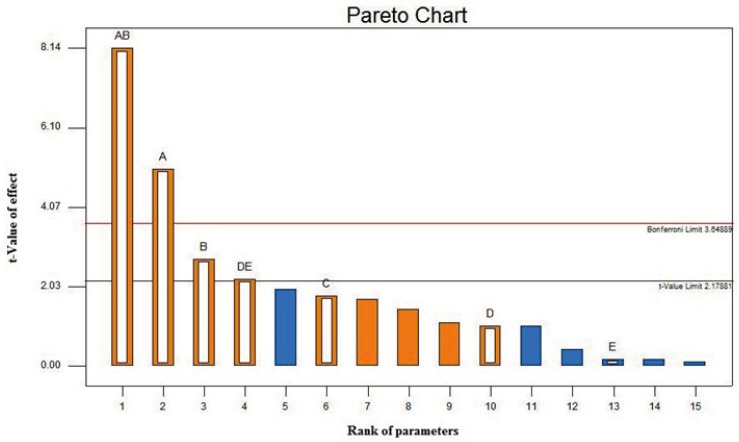
Pareto chart of dA at 95% confidence interval. A, B, C, D and E are concentration, pH, temperature, feed rate and gas atomizing flow rate respectively.

The Pareto chart of the main and interaction effects is shown in Figure 1. The Pareto chart is used to identify experimental parameters that have a statistically significant influence on a particular response. The Pareto chart displays the magnitude of the effects and draws a reference line at a 95% confidence level. Effects with a magnitude that extends beyond the reference line are statistically significant(43). The Pareto chart for dA reveals that the significant spray drying formulation parameters are the feed concentration, the feed pH, and the interaction between the feed rate and the gas atomizing flow rate. The inlet temperature, which is known to significantly influence dA of spray dried particles, is found to have a reduced impact on the nanoaggregate production. The effect of the inlet temperature selection on dA is insignificant provided that the selected value can adequately provide a drying rate to produce the nanoaggregates.

The Pareto chart also indicates that strong interactions exist between the feed concentration and the pH and between the feed rate and the gas atomizing flow rate. The strong interaction between the feed concentration and the pH indicates that the effect of varying the feed concentration on dA is dependent on the pH value and vice versa. Significant effect of concentration, pH and interaction between them, may be occurred because of the high pH dependent stability of colloidal silica (34, 44). The strong interaction between the feed rate and the gas atomizing flow rate is attributed to the fact that spray drying at a higher feed rate (F) must likely be accompanied by an increase in the gas atomizing flow rate (A) to prevent an excessive wetting of the drying chamber, which can lead to a lower production yield and higher particle moisture content. A higher A/F ratio indicates a stronger atomization force resulting in smaller size droplets that generally lead to smaller size of the spray-dried particles.

In summary, the screening design has taught us that the feed concentration, the feed pH, and the ratio of the gas atomizing flow rate to the feed rate (*i.e*. A/F ratio) are the three spray-drying formulation parameters that govern dA of the nanoaggregates. The next step is to optimize those parameters to produce nanoaggregate with particle size in range of 2-4 µm by employing a response surface method.

*Optimization of spray drying process*

In order to achieve nanoaggregates with dA between 2-4 µm, a response surface method was used. Box-Behnken design is an experimental design for achieving a quadratic model, which explain the relation between independent variables and response (45) and by solving the obtained quadratic equation, magnitudes of independent variable which result to desirable response, will be obtained.

The Box-Behnken method was applied on the independent variables which had a significant effect on the dA. Concentration of the feed, pH of the feed and the ratio of gas atomizing flow rate to the feed rate (A/F) were the significant factors based on the screening test. The levels of these factors chose broader to cover more conditions. Temperature was kept on the 100 °C (center point of screening test). In Table 2 these factors and their levels are shown.

**Table 2:** Factors and their magnitude used in Box-Behnken design

**Factor**	**Name**	**Units**	**Type**	**Low actual**	**High actual**	**Low coded**	**High coded**
A	Concentration	(w/w%)	Numeric	2	4	-1	1
B	pH		Numeric	5	9	-1	1
C	T	°C	Numeric	90	110	-1	1
D	Feed	mL/min	Numeric	1	2	-1	1
E	Air flow	m^3^/h	Numeric	0.34	0.38	-1	1

The 22 experimental runs of the half-factorial design and their responses are presented Table 3. The results in Table 3 are the average of the two independent replicates.

**Table 3:** Summary of Box-Behnken design

**Run**	**Block**	**Factor 1**	**Factor 2**	**Factor 3**	**Response**
		**A:Concentration** **(w/w%)**	**B:pH**	**C:A/F ratio**	**dA** **µm**
1	Week 1	4.6	10.4	0.26	5.6
2	Week 1	3	10.4	0.18	3.5
3	Week 1	1.4	7	0.34	4.5
4	Week 1	3	10.4	0.34	3.9
5	Week 1	1.4	3.6	0.26	3.7
6	Week 1	4.6	3.6	0.26	4.1
7	Week 1	4.6	7	0.18	4.9
8	Week 1	3	7	0.26	3.4
9	Week 1	1.4	10.4	0.26	4.1
10	Week 1	3	7	0.26	3.6
11	Week 1	4.6	7	0.34	4.8
12	Week 1	3	7	0.26	3.8
13	Week 1	1.4	7	0.18	3.5
14	Week 1	3	3.6	0.34	3.4
15	Week 1	3	3.6	0.18	3.5

The Sequential model sum of squares and the Lack of fit tests showed that the quadratic model is the best model for fitting the response in the range of independent variables. The results of these tests are shown in Table 4 and in Table 5 respectively.

**Table 4:** Sequential model sum of squares for Box-Behnken designs

**Sum of** **source**	**Mean** **squares**	**df**	**Square**	**f-value**	**p-value** **Prob > F**
Mean vs total	242.41	1	242.41		
Linear vs mean	2.52	3	0.84	2.62	0.1031
2FI vs linear	0.67	3	0.22	0.62	0.6197
Quadratic vs 2FI	2.48	3	0.83	11.03	0.0121
Cubic vs quadratic	0.29	3	0.098	2.46	0.3023

**Table 5:** Lack of fit tests for Box-Behnken design

**Source**	**Sum of** **squares**	**df**	**Mean** **square**	**f-value**	**p-value** **Prob > F**
Linear	3.44	9	0.38	9.57	0.0982
2FI	2.78	6	0.46	11.57	0.0817
Quadratic	0.29	3	0.098	2.46	0.3023

**Figure 2:**
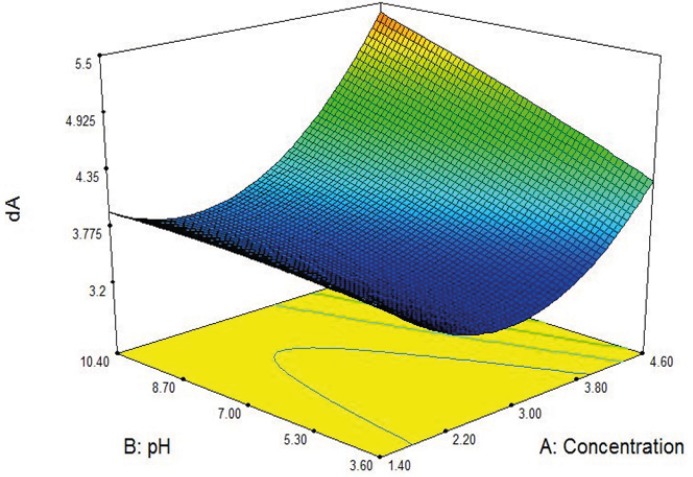
Effect of pH feed and concentration feed on the dA.

In Figure 2 effects of feed pH and feed concentration on the dA are shown in a 3D graph. As it can be seen there is optimum point for dA magnitude.

The final equation in terms of coded factors is shown in Table 6.

**Table 6:** Quadratic regression equation for the response surface design

**Response**	**Constant**	**A**	**B**	**C**	**AB**	**AC**	**BC**	**A** ^2^	**B** ^2^	C^2^
dA	+4.0702	-1.418	-0.137	+4.0878	+0.051	-2.148	+0.460	+0.317	-3.243	+1.953

dA: aerodynamic diameter

A: feed concentration (w/w %)

B: feed pH

C: feed rate (m^3^/h)

The software offers several solutions for the quadratic equation in order to obtain a dA in range 3.4 to 4 µm, one of them was selected, and nanoaggregates were prepared under that condition in laboratory with spray dryer. The average of dA of the five independent replicates was compared to the calculated dA from the equation and the result showed 95% of accuracy in particle size.

The *in-vitro* evaluations were performed on these batches of nanoaggregates. 

*In-vitro evaluation of rifampin-loaded silica nanoaggregates*

*SEM*

**Figure 3:**
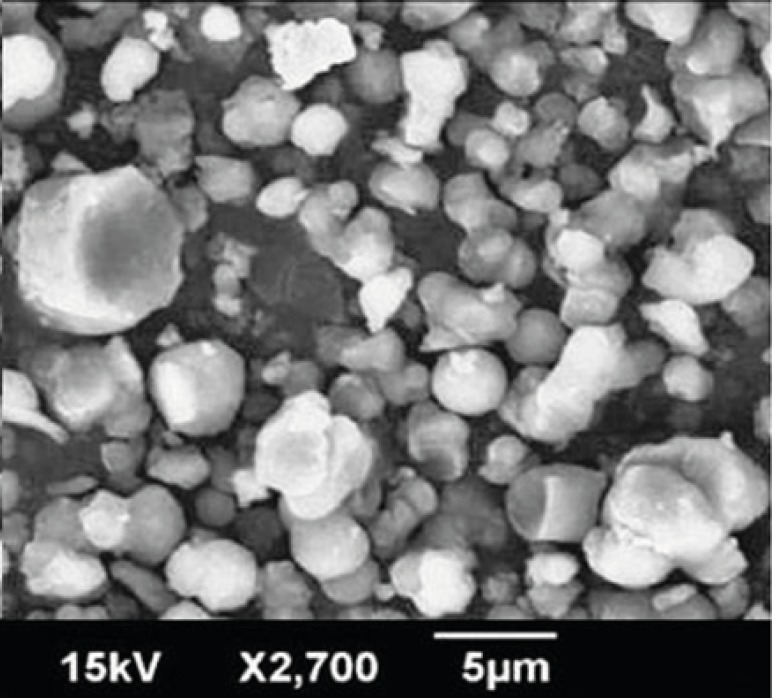
SEM of Rifampin-loaded silica nanoaggregates

Figure 3 displays scanning electron microscopy observations of Rifampin-loaded silica nanoaggregates. Nanoaggregates were observed as spherical particles with a size of about 5 µm.

***Particle size measurements***

**Figure 4:**
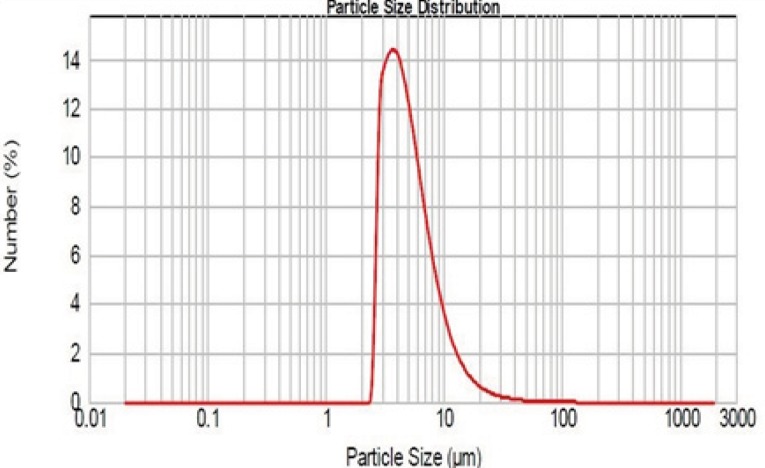
Size distribution of Rifampin-loaded silica nanoaggregates.

As mentioned, the particle size was determined with Mastersizer 2000. The results obtained are shown in Figure 4.

As it can be seen, the logarithmic particle size distribution is normal and geometric mean diameter is 4.64 ± 0.9 µm (n=3, mean±SD). D_10_ and D_90_ are 2.97 and 9.76 µm respectively. The size and sharpness of the peak indicate that the Rifampin-loaded nanoaggregates are suitable for pulmonary drug delivery. 

The densitometry analysis of Rifampin-loaded nanoaggregates showed that the tapped density of them is 0.48 ± 0.05 g/cm^3^ (n=3, mean±SD). for calculation of effective density, the tapped density should be correct by a factor of 0.79^-1^ for taking into account the imperfect packing after tapping (40). The magnitude of effective density (ρ_eff_) was equal to 0.6 g/cm^3^. By using the Equation (1) the magnitude of dA was equal to 3.59 ± 0.7 µm which is in suitable range for pulmonary drug delivery.

*In-vitro drug release*

**Figure 5:**
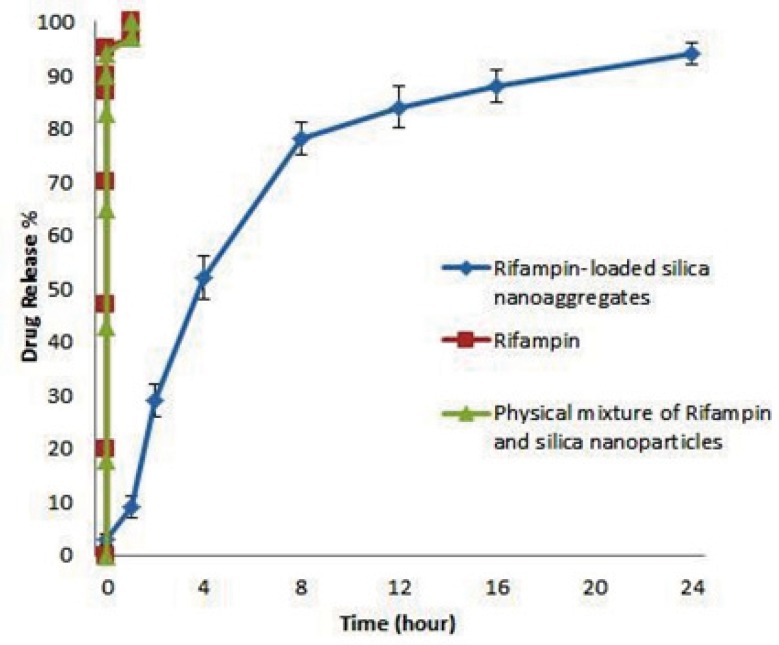
Release profile of Rifampin, physical mixture of Rifampin and nanoparticles and Rifampin-loaded silica nanoaggregates in pH 7.4 phosphate buffer medium at 37 °C (n=3, mean±SD).

Release profile of Rifampin-loaded silica nanoaggregate, Rifampin and physical mixture of Rifampin and silica nanoparticles were investigated in phosphate buffer as the test medium. Accurately weighed amounts of the prepared sample was used under sink conditions (C < 0.2Cs). The obtained results are shown in Figure 5.

As it can be seen Rifampin and physicals mixture of Rifampin and silica nanoparticle achieved a 100% of release in about 1 hour, but interestingly Rifampin-loaded nanoaggregates showed a triphasic release pattern and released 90% of drug after 24 hours.

First phase of release was in first hour of test in which the rate of release is slower than other 2 phase, this phenomena may be caused by the time that nanoaggregates need to re-disperse. The lower rate of release could be explained by the greater surface area of nanoaggregates.

Second phase of release which happened from 1 to 8 hours has the most rate of release. Fitting the data of this phase with equation of release pattern of spherical particles (46) showed an adjusted R square of 0.994. By considering that the initial silica nanoparticles were spherical (27), may be the second phase of release caused by diffusion of rifampin molecules from the spherical nanoparticles.

Third phase of release which happened from 8 to 24 hours showed a zero order of release.

*Aqueous re-dispersibility characterizations*

**Figure 6:**
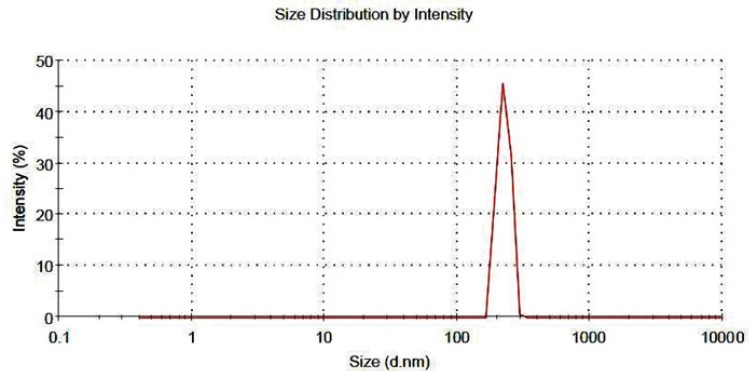
particle size distribution of re-dispersed nanoaggregates.

The nanoaggregates must readily re-disperse into the primary nanoparticles in an aqueous medium for the nanoparticles to perform their intended therapeutic functions. The result of this test showed that the diameter of nanoaggregates decreased up to 80% after the gentle stirring. The particle size distribution of the re-dispersed nanoaggregates is shown in Figure 6, as it can be seen all of nanoaggregates dispersed in the medium after 30 minute of gentle stirring. This results indicate that re-dispersibility of the nanoaggregates happen in a reasonable time and the initial nanoparticles could release the loaded-drug in order to perform the therapeutic function (42).

*Aerosol performance of Rifampin-loaded nanoparticles*

The results of this test showed that the fine particle fraction (FPF) and the emitted dose are 46.12 ± 0.6 and 82.7 ± 4.5 % respectively. The FPF is the most important parameter to evaluate *in-vitro* performance of the aerosols and its magnitude varies in wide range because many factors affect the aerosolization of the powders. But in most cases a FPF of about 50 % is a good magnitude for an acceptable deposition in the lung (47).

## Conclusion

The results obtained showed that spray dryer could produce suitable nanoaggregates for pulmonary drug delivery. The dA of nanoaggregates is influenced by feed concentration, feed pH and interaction of feed flow rate and gas atomizer flow rate. Re-dispersability test showed that the rifampin-loaded nanoaggregates could convert to nanoparticles form in order to release drug content in lungs. The aerosol performance test showed that about 50% of nanoaggregates could deposit in lungs.

Drug release studies showed Rifampin-loaded silica nanoaggregates could be release the rifampin within 24 hours up to 90% with a combination patterns of release. A prolonged release of rifampin molecules included within mesopores principally by a diffusion phenomenon. Hence, it seems that association of a nanostructured mineral to the molecular state of the drug presents a great interest for pharmaceutical applications, as it allows control over the kinetics of drug delivery, especially for lipophilic drugs. 
